# Supplementary material for: Detection of isoforms and genomic alterations by high-throughput full-length single-cell RNA sequencing in ovarian cancer
Source: Nat Commun. 2023 Nov 27;14:7780. doi: 10.1038/s41467-023-43387-9 (PMC10682465; doi:10.1038/s41467-023-43387-9)
Supplement: Supplementary file 1 — Supplementary Information [file 41467_2023_43387_MOESM1_ESM.pdf]

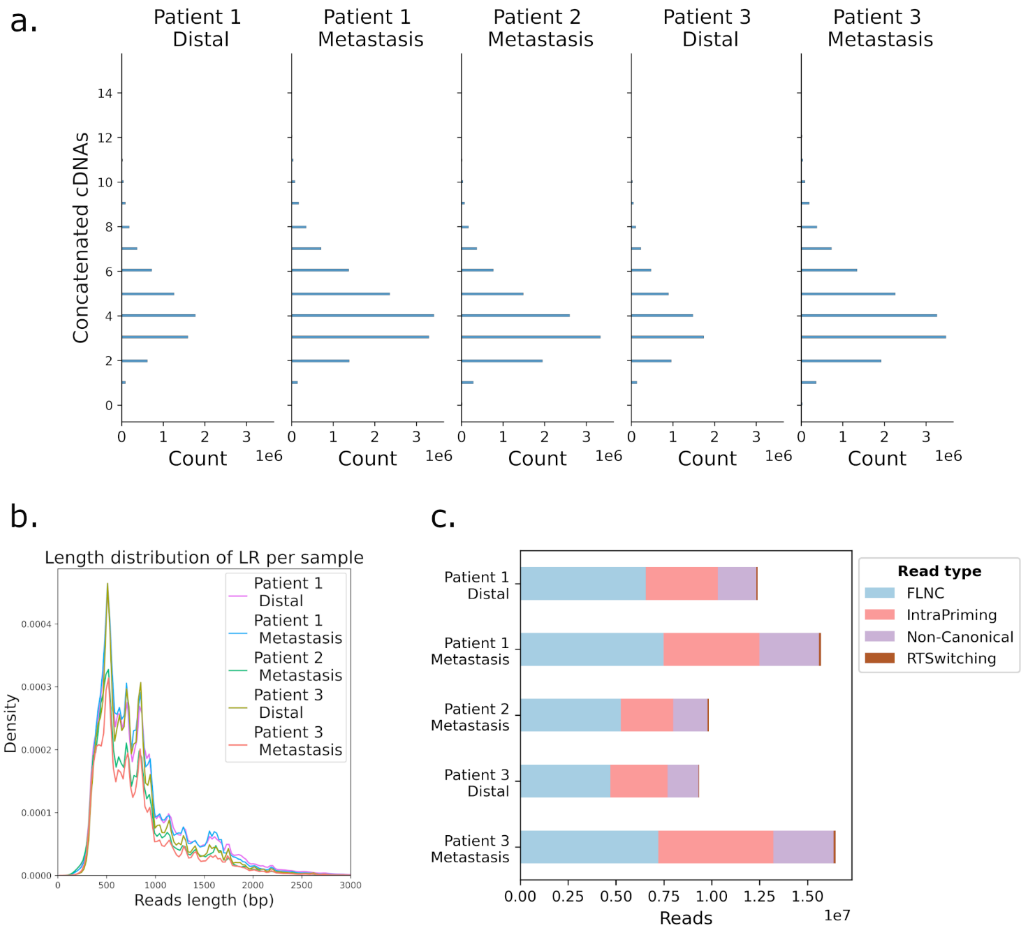

Supplementary Figure 1: QC metrics of long reads. **(a)** Histogram showing the number of concatemeric cDNA molecules per sequencing read by sample. **(b)** Length distribution of long reads after unconcatenation, per sample. **(c)** Number of UMIs belonging to isoforms passing all filters (full-length non-chimeric) and UMIs belonging to filtered isoforms (intra-priming, reverse transcriptase (RT) switching, non-canonical isoforms), following SQANTI classification.

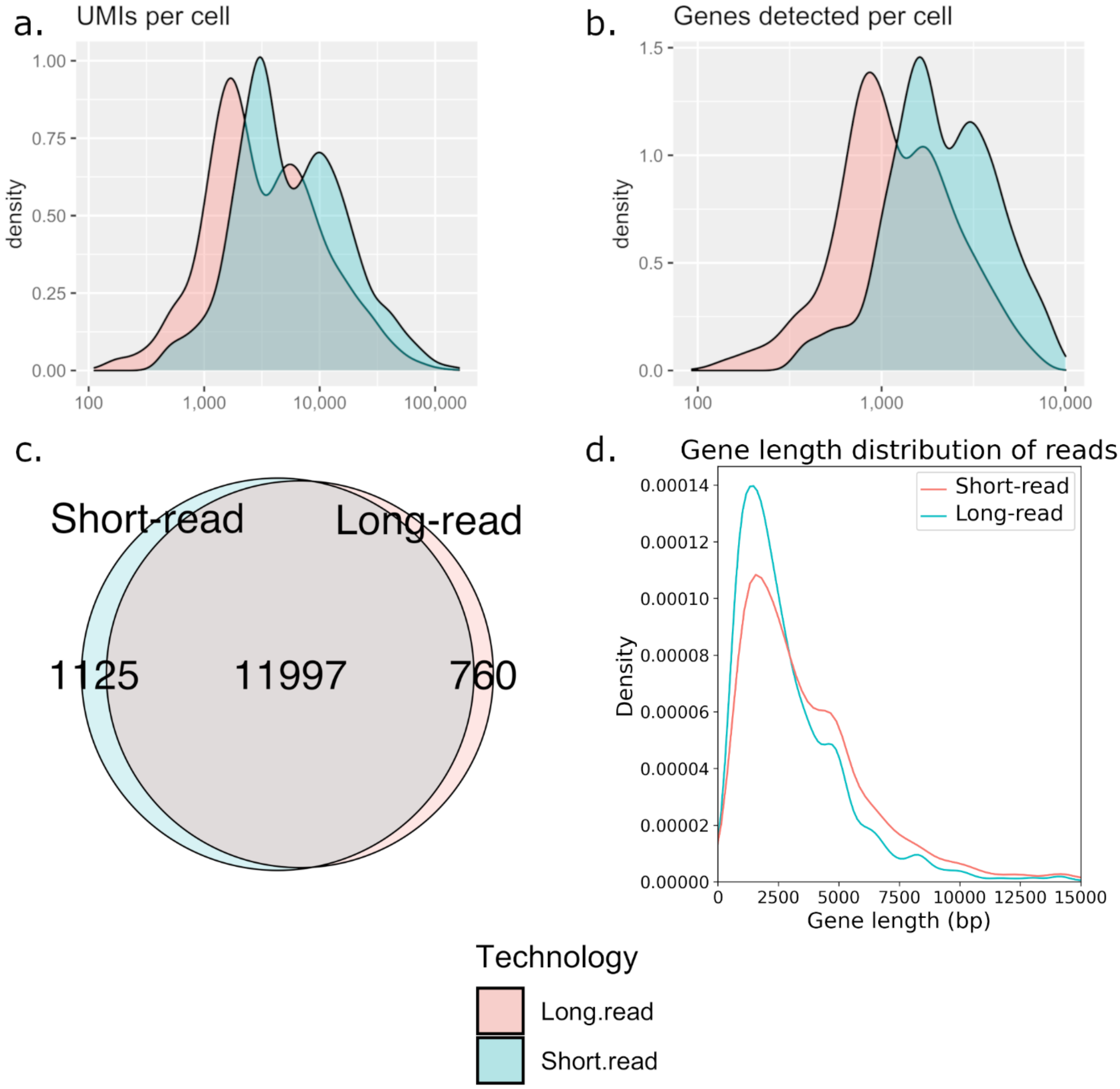

Supplementary Figure 2: QC metrics comparison of short and long reads. **(a)** Distribution of single UMI reads and **(b)** genes detected per cell in short- (light-blue) and long-read (light-red) technologies. **(c)** Overlap of total detected genes between short- and long-read datasets. **(d)** Gene length distribution of genes detected in short- and long-read technologies, where gene length equals the sum of exon length of the longest isoform associated with the gene.

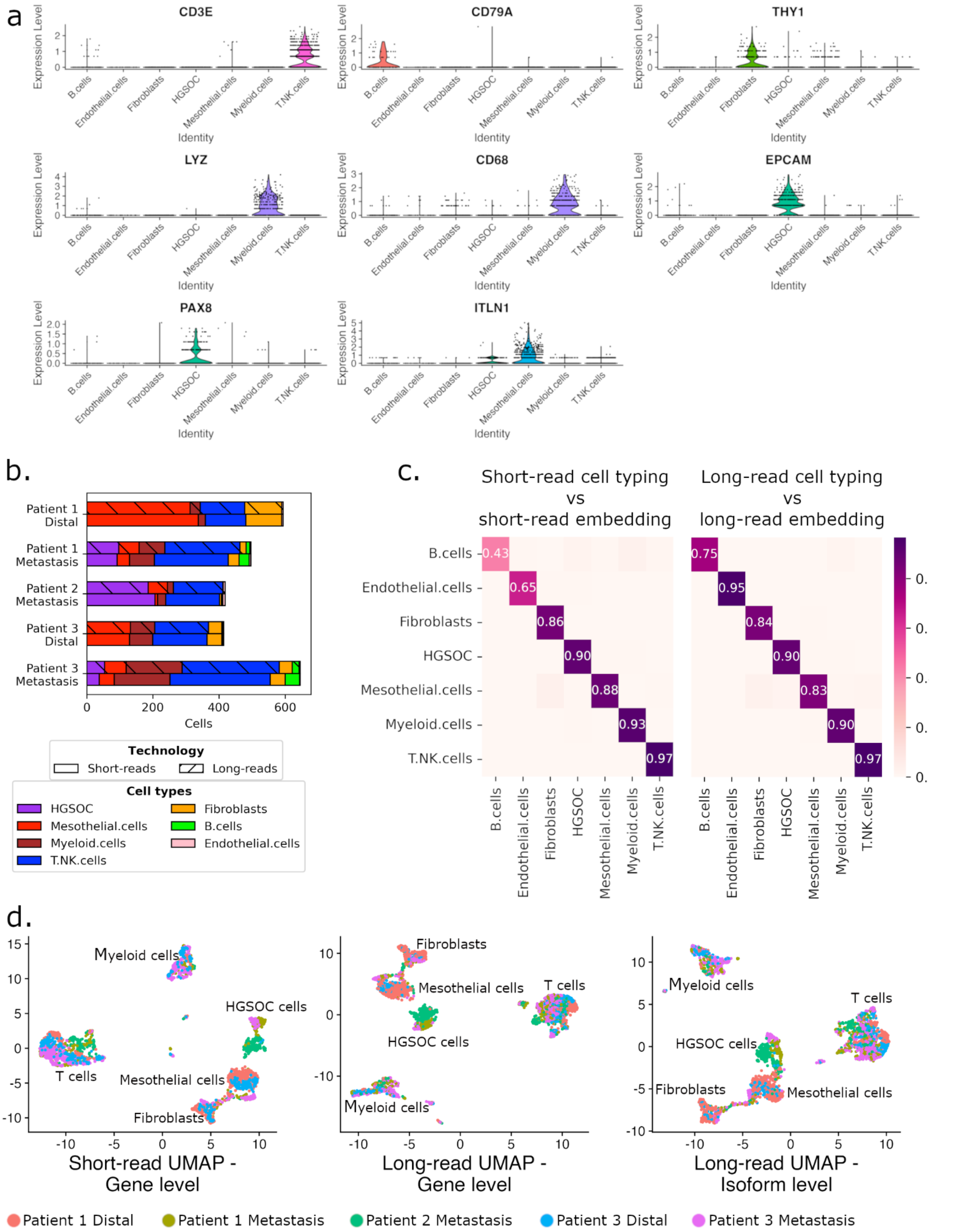

Supplementary Figure 3: Cell type marker detection and embedding by sample. (a) Violin plot showing expression of marker genes in long-read data. CD3E is a T cell marker, CD79A a B cell marker, THY1 a fibroblast marker, LYZ and CD68 myeloid cell markers, EPCAM and PAX8 are HGSOC markers and ITLN1 a mesothelial cell marker. **(b)** Cell type composition bar plot per sample per sequencing technology. **(c)** Jaccard distance of cell populations in different UMAP embeddings: short-read cell labeling versus short-read UMAP embedding (left), and long-read cell labeling versus long-read UMAP embedding. **(d)** UMAP cohort visualization by sample ID for short-read - gene level (left), long-read - gene level (middle), and long-read - transcripts level (right) data.

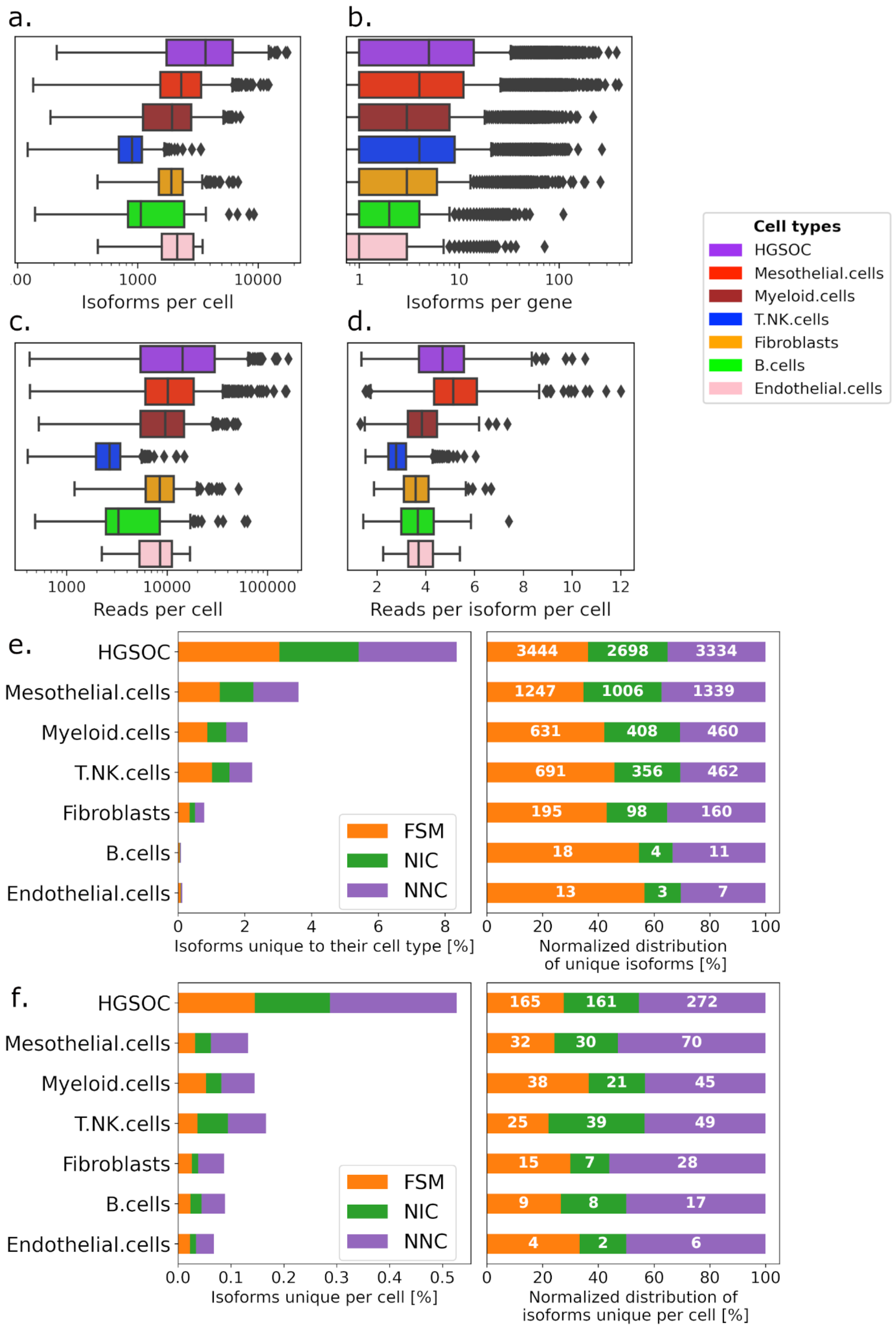

Supplementary Figure 4: QC metrics by cell type in long-read sequencing. (a) Number of isoforms detected per cell, by cell type. **(b)** Number of isoforms detected per gene, by cell type. **(c)** Number of unique reads per cell in long-reads, by cell type. **(d)** Long reads per isoform in each cell, by cell type. Boxes display the first to third quartile with median as horizontal line, whiskers encompass 1.5 times the interquartile range, and data beyond that threshold is indicated as outliers. **(e)** Percentage of isoforms detected per cell type that are unique to this particular cell type (left panel), and their SQANTI-defined structural category normalized distribution (right panel, number of isoforms displayed in white). **(f)** Percentage of isoforms detected per cell type that are only found in one cell (left panel), and their SQANTI-defined structural category normalized distribution (right panel, number of isoforms displayed

in white).

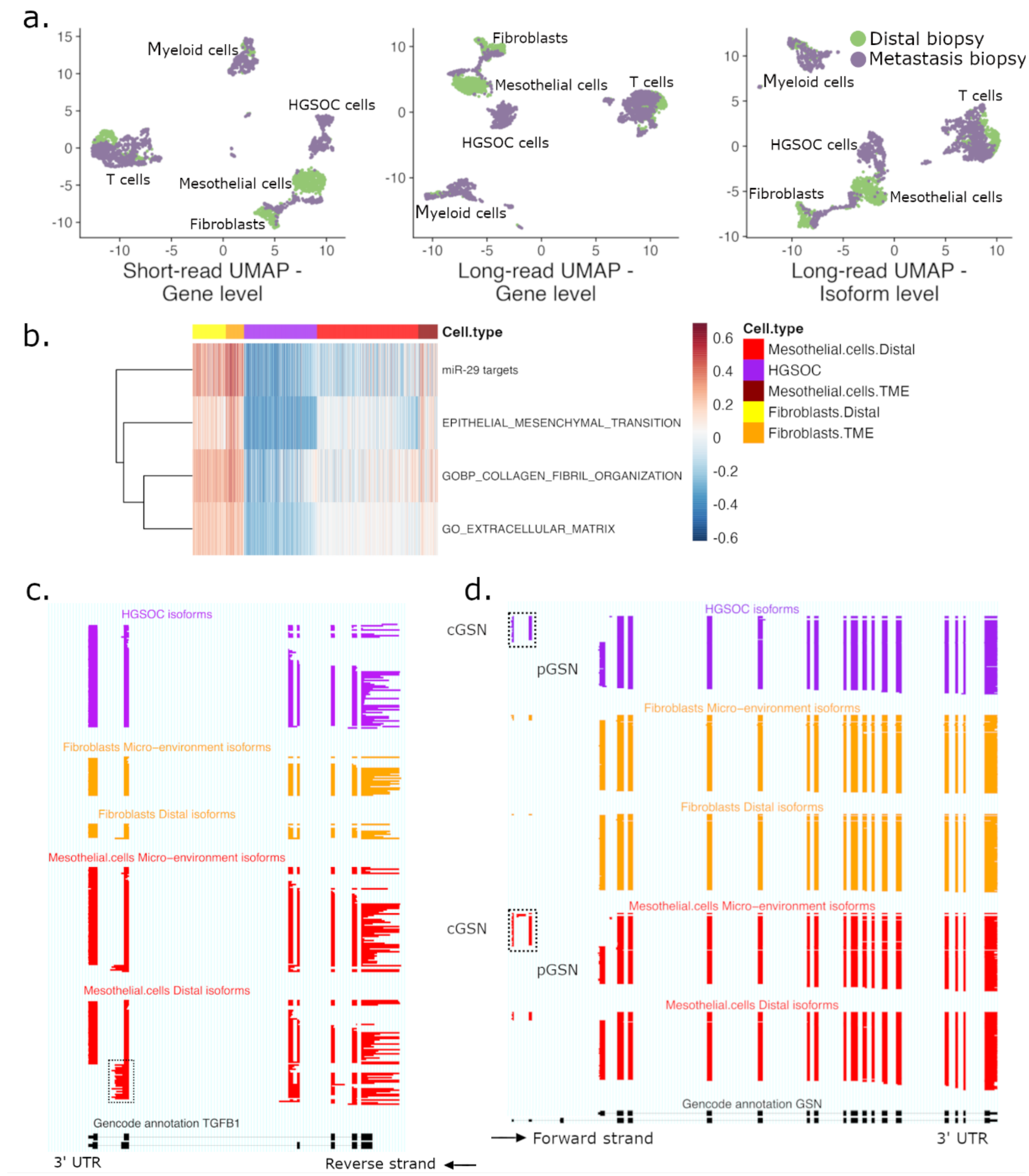

Supplementary Figure 5: Epithelial-to-mesenchymal transition in the tumor microenvironment. **(a)** UMAP embeddings of the cohorts' long-read data of short-read data - gene level (left), long-read data - gene level (middle), long-read data - isoform level (right), colored by tissue type. **(b)** Gene set variation analysis (GSVA) scores for different cell types. Heatmap colors from blue to red represent low to high enrichment. **(c)** ScisorWiz representation of isoforms in *TGFB1*. Dashed box highlights the non-canonical 3'UTR used by distal mesothelial cells. **(d)** ScisorWiz representation of isoforms in *GSN*. Dashed boxes highlight the TSS, where mesothelial TME and HGSOC cells differentially express the *cGSN* isoform, while mesothelial distal cells and fibroblasts use *pGSN*.

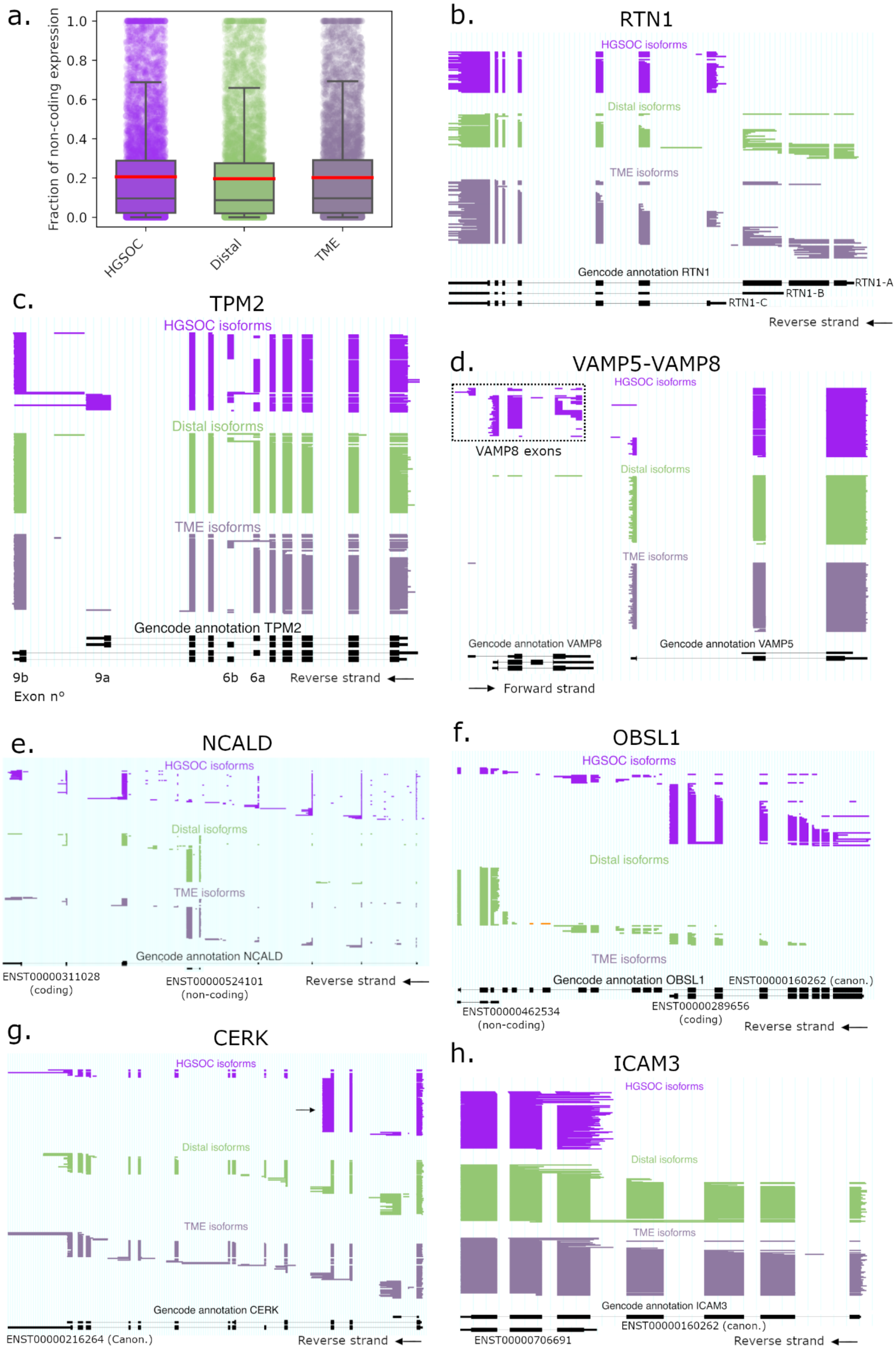
a.
Fraction of non-coding expression
1.0
0.8
0.6
0.4
0.2
0.0
HGSOC
Distal
TME
b.
RTN1
HGSOC isoforms
Distal isoforms
TME isoforms
Gencode annotation RTN1
RTN1-A
RTN1-B
RTN1-C
Reverse strand
c.
TPM2
HGSOC isoforms
Distal isoforms
TME isoforms
Gencode annotation TPM2
9b
9a
6b
6a
Reverse strand
Exon n°
d.
VAMP5-VAMP8
HGSOC isoforms
VAMP8 exons
Distal isoforms
TME isoforms
Gencode annotation VAMP8
Gencode annotation VAMP5
Forward strand
e.
NCALD
HGSOC isoforms
Distal isoforms
TME isoforms
Gencode annotation NCALD
ENST00000311028
(coding)
ENST00000524101
(non-coding)
Reverse strand
f.
OBSL1
HGSOC isoforms
Distal isoforms
TME isoforms
Gencode annotation OBSL1
ENST00000160262 (canon.)
ENST00000462534
(non-coding)
ENST00000289656
(coding)
Reverse strand
g.
CERK
HGSOC isoforms
Distal isoforms
TME isoforms
Gencode annotation CERK
ENST00000216264 (Canon.)
Reverse strand
h.
ICAM3
HGSOC isoforms
Distal isoforms
TME isoforms
Gencode annotation ICAM3
ENST00000160262 (canon.)
ENST00000706691
Reverse strand

Supplementary Figure 6: Top differentially expressed isoforms between HGSOC and all distal cells.

(a) Boxplot of the fraction of non-coding expression in protein-coding genes, where each point is a protein-coding gene**.** Boxes display the first to third quartile with median as horizontal black line, mean as horizontal red line, whiskers encompass 1.5 times the interquartile range, and data beyond that threshold is indicated as outliers. ScisorWiz representation of isoforms in **(b)** *RTN1*, **(c)** *TPM2*, **(d)** *VAMP5-VAMP8*, **(e)** *NCALD*, **(f)** *OBSL1*, **(g)** *CERK*, and **(h)** *ICAM3* in cancer, all distal, and all TME cells. Each horizontal line represents a single isoform colored according to cell types. Notable reference isoforms from GENCODE are named on the bottom of each gene.

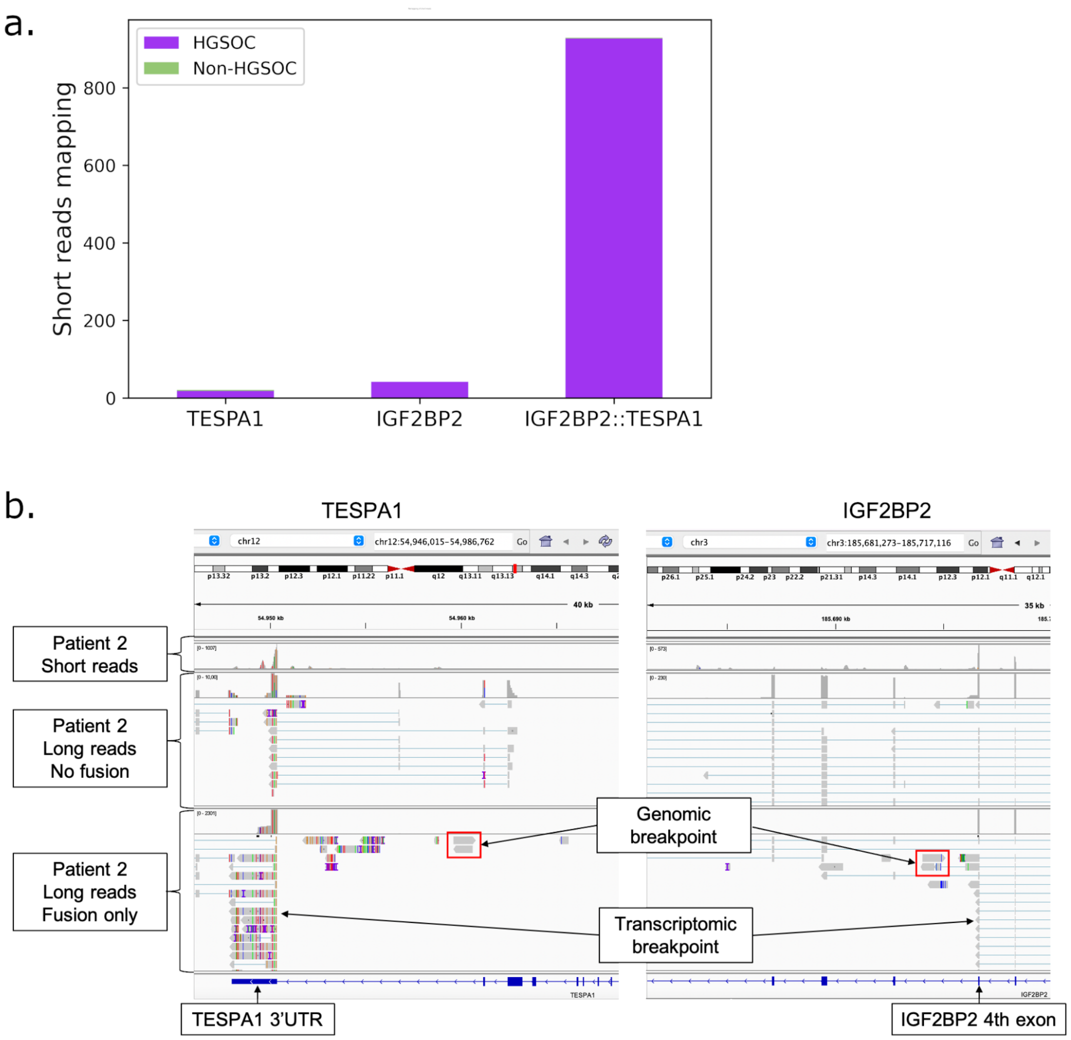

Supplementary Figure 7: Transcriptomic and genomic breakpoint validation of the *IGF2BP2::TESPA1* gene fusion. **(a)** Remapping of scRNA short-reads previously mapping to *IGF2BP2* or *TESPA1* and overlapping the fusion breakpoint. Most reads are preferentially remapped to the *IGF2BP2::TESPA1* transcript template. **(b)** Overview of *IGF2BP2::TESPA1* gene fusion genomic and transcriptomic breakpoints, on IGV genome browser. The two intronic fusion long reads in the red box share the same breakpoint and were used as template for the genomic fusion breakpoint reference. The transcriptomic breakpoint occurs on the closest 5' exon junction of the genomic breakpoint in *IGF2BP2*, and on the closest 3' exon junction in *TESPA1*.

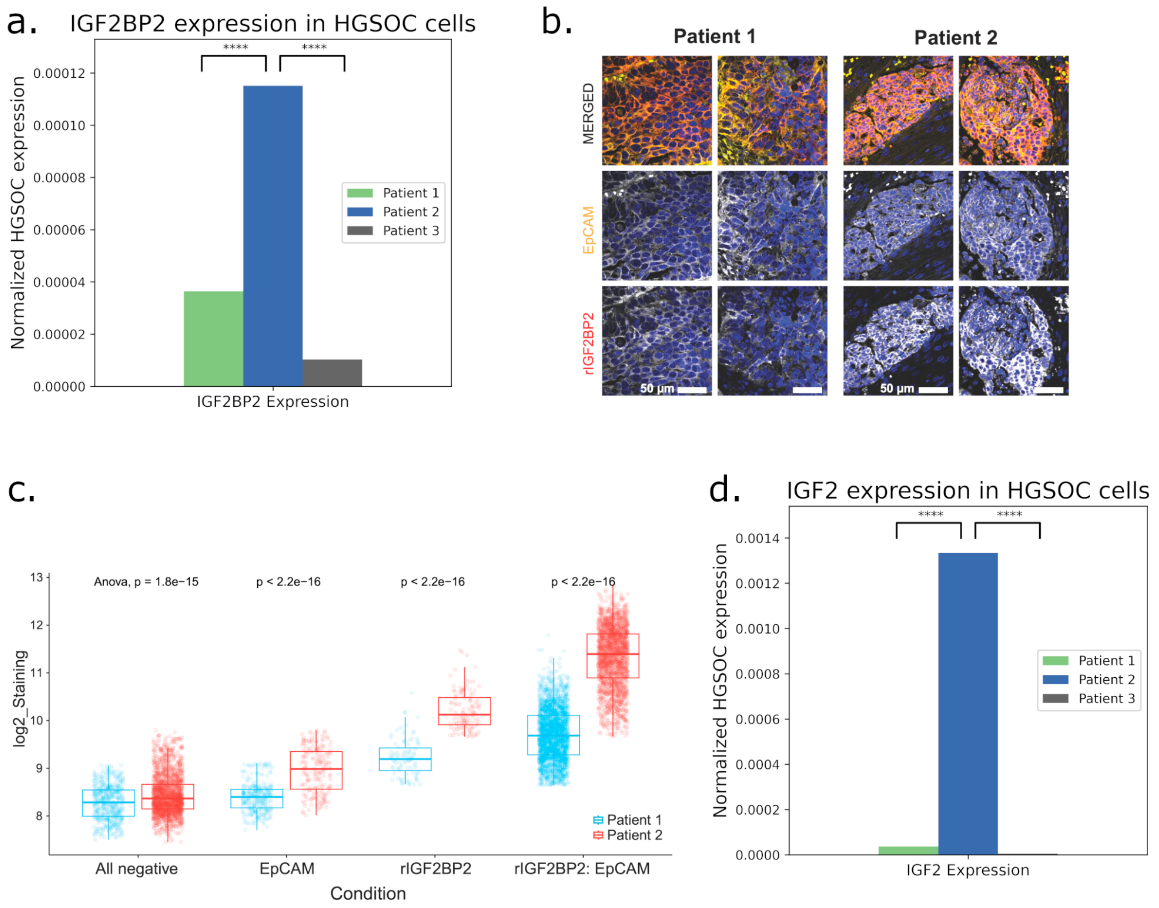

Supplementary Figure 8: Immunofluorescence validation of IGF2BP2 expression.
(a) Normalized expression of IGF2BP2 in cancer cells, per patient. **(b)** Representative immunofluorescence images (two tissue regions per patient) for detection of tumor (EpCAM$^+$) and C-terminal IGF2BP2 expressing cells in Patient 1 (no fusion) and Patient 2 harboring the *IGF2BP2::TESPA1* fusion. Patients = 2, Replicates per patient = 2. Source images are provided as Source Data files. **(c)** Quantification of immunofluorescence images for determination of cancer cell-specific expression of IGF2BP2 in matched patient tissue samples. **(d)** Normalized expression of IGF2 in cancer cells, per patient

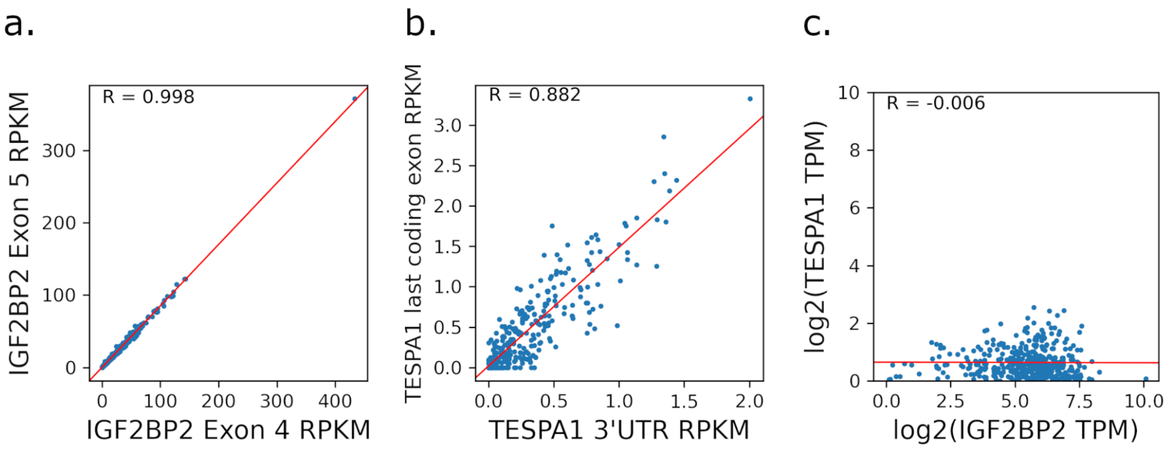

Supplementary Figure 9: **Detection of the *IGF2BP2::TESPA1* fusion in the ovarian cancer TCGA data.**

(a) TCGA expression of *IGF2BP2* exons 4 and 5, surrounding the *IGF2BP2::TESPA1* breakpoint. **(b)** TCGA expression of *TESPA1* last coding exon and 3'UTR exon, surrounding the *IGF2BP2::TESPA1* breakpoint. **(c)** Log2 normalized gene expressions of *IGF2BP2* and *TESPA1* in TCGA.

Supplementary Table 1: Number of long reads per pre-processing step. Full-length non-chimeric (FLNC) reads are the reads attached to isoforms passing all SQANTI filters.

Patient	Biopsy site	#SMRT 8M cells	# cells	Sequencing output	Unconcatenated	Barcoded & poly-adenylated	Unique UMIs	FLNC	Av. reads/cell
Patient 1	Distal omentum	2	594	6,779,153	29,544,748	27,700,151	12,406,703	6,554,277	11034
Patient 1	Metastasis omentum	4	497	13,384,311	57,548,787	53,882,454	15,705,384	7,033,927	14152
Patient 2	Metastasis omentum	4	419	6,121,676	23,941,842	53,882,454	9,853,727	5,259,277	12551
Patient 3	Distal omentum	2	415	14,110,642	58,681,499	22,312,230	9,358,572	4,698,768	11322
Patient 3	Metastasis omentum	4	646	11,127,247	41,972,301	38,804,145	16,481,265	7,198,920	11143
Total	/	16	2571	51,523,029	211,689,177	196,581,434	63,805,651	30,745,169	/

Supplementary Table 2: Number of short reads per pre-processing step. scAmpi output includes the filtering of non-coding, mitochondrial and ribosomal genes.

**Patient**	**Biopsy site**	**# cells**	**Sequencing output**	**scAmpi output**
Patient 1	Distal omentum	594	36,906,084	4,965,329
Patient 1	Metastasis omentum	497	34,956,064	4,619,179
Patient 2	Metastasis omentum	419	69,122,342	4,450,169
Patient 3	Distal omentum	415	101,669,266	7,290,225
Patient 3	Metastasis omentum	646	94,921,371	4,988,124
Total	/	2571	337,575,127	26,313,026
