# Supplementary material for: Detection of isoforms and genomic alterations by high-throughput full-length single-cell RNA sequencing in ovarian cancer
Source: Nat Commun. 2023 Nov 27;14:7780. doi: 10.1038/s41467-023-43387-9 (PMC10682465; doi:10.1038/s41467-023-43387-9)
Supplement: Supplementary file 3 — Description of Additional Supplementary Files [file 41467_2023_43387_MOESM3_ESM.pdf]

Description of Additional Supplementary Files

File Name: Supplementary Data 1
Description: Table of mutated cells and the somatic or germline mutations they harbor.

File Name: Supplementary Data 2
Description: Differential gene expression between TME and Distal Mesothelial cells.

File Name: Supplementary Data 3
Description: Genes predicted by miRDB to be targeted by the miR-29 family.

File Name: Supplementary Data 4
Description: Differential isoform usage between HGSOC and distal cells.

File Name: Supplementary Data 5
Description: Table of detected fusions containing more than 10 UMIs.
