# Supplementary material for: Detection of isoforms and genomic alterations by high-throughput full-length single-cell RNA sequencing in ovarian cancer
Source: Nat Commun. 2023 Nov 27;14:7780. doi: 10.1038/s41467-023-43387-9 (PMC10682465; doi:10.1038/s41467-023-43387-9)
Supplement: Supplementary file 9 — Reporting Summary [file 41467_2023_43387_MOESM9_ESM.pdf]

nature portfolio

Corresponding author(s): Niko Beerenwinkel

Last updated by author(s): Oct 23, 2023

Reporting Summary

Nature Portfolio wishes to improve the reproducibility of the work that we publish. This form provides structure for consistency and transparency in reporting. For further information on Nature Portfolio policies, see our Editorial Policies and the Editorial Policy Checklist.

Statistics

For all statistical analyses, confirm that the following items are present in the figure legend, table legend, main text, or Methods section.

n/a	Confirmed	
[ ]	[x]	The exact sample size (*n*) for each experimental group/condition, given as a discrete number and unit of measurement
[ ]	[x]	A statement on whether measurements were taken from distinct samples or whether the same sample was measured repeatedly
[ ]	[x]	The statistical test(s) used AND whether they are one- or two-sided<br>*Only common tests should be described solely by name; describe more complex techniques in the Methods section.*
[ ]	[x]	A description of all covariates tested
[ ]	[x]	A description of any assumptions or corrections, such as tests of normality and adjustment for multiple comparisons
[x]	[ ]	A full description of the statistical parameters including central tendency (e.g. means) or other basic estimates (e.g. regression coefficient) AND variation (e.g. standard deviation) or associated estimates of uncertainty (e.g. confidence intervals)
[ ]	[x]	For null hypothesis testing, the test statistic (e.g. *F*, *t*, *r*) with confidence intervals, effect sizes, degrees of freedom and *P* value noted<br>*Give P values as exact values whenever suitable.*
[x]	[ ]	For Bayesian analysis, information on the choice of priors and Markov chain Monte Carlo settings
[x]	[ ]	For hierarchical and complex designs, identification of the appropriate level for tests and full reporting of outcomes
[x]	[ ]	Estimates of effect sizes (e.g. Cohen's *d*, Pearson's *r*), indicating how they were calculated

Our web collection on statistics for biologists contains articles on many of the points above.

Software and code

Policy information about availability of computer code

Data collection

SMRT-Link: https://www.pacb.com/support/software-downloads/ v9.0.0.92188

Data analysis

Tools deigned for analysis:
Wrapper code used to pre-process and analyze the data: https://github.com/cbg-ethz/scIsoPrep.
Long-reads 3'UTR analysis: https://github.com/ArthurDondi/DaPars2_LR

Tools used for analysis can be found below:
BBLAST: https://ftp.ncbi.nlm.nih.gov/blast/executables/blast+/2.5.0/ v2.5.0
bwa: https://github.com/lh3/bwa v0.7.17
cDNA_cupcake: https://github.com/Magdoll/cDNA_Cupcake/releases/tag/v28.0.0 v28.0.0
CellRanger: https://support.10xgenomics.com/single-cell-gene-expression/software/release-notes/3-1 v3.1
cellranger-dna: https://support.10xgenomics.com/single-cell-dna/software/downloads/latest v1.1.0
IsoSeq https://github.com/PacificBiosciences/IsoSeq v3.4
minimap2: https://github.com/lh3/minimap2 v2.17
scAmpi: https://github.com/ETH-NEXUS/scAmpi_single_cell_RNA v1.0
SCICoNE https://github.com/cbg-ethz/SCICoNE
Scisorseqr: https://github.com/noush-joglekar/scisorseqr v0.1.2
ScisorWiz https://github.com/ans4013/ScisorWiz
SMRT-Link: https://www.pacb.com/support/software-downloads/ v9.0

SQANTI3 https://github.com/ConesaLab/SQANTI3/archive/refs/tags/v1.6.tar.gz v1.6

For manuscripts utilizing custom algorithms or software that are central to the research but not yet described in published literature, software must be made available to editors and reviewers. We strongly encourage code deposition in a community repository (e.g. GitHub). See the Nature Portfolio guidelines for submitting code & software for further information.

Data

Policy information about availability of data

All manuscripts must include a data availability statement. This statement should provide the following information, where applicable:

- Accession codes, unique identifiers, or web links for publicly available datasets
- A description of any restrictions on data availability
- For clinical datasets or third party data, please ensure that the statement adheres to our policy

The raw sequencing files, as well as the associated analysis files (isoforms gff file, reads associated to isoforms, and the Gencode annotation of novel isoforms) reported in this study have been deposited in the European Genome-phenome Archive (EGA) under the accession number EGAS00001006807 ((https://ega-archive.org/studies/EGAS00001006807). They are available under restricted access in compliance with data privacy laws. Patient 2 scDNA reads mapping the IGF2BP2::TESPA1 fusion breakpoint are available at https://eth-nexus.github.io/tu-pro_website/publications/dondi_et_al_2022/. The TCGA data was obtained at https://portal.gdc.cancer.gov/repository, with the following specifications: Project=TCGA-OV, DataType=Gene Expression Quantification. Human genome hg38 is available at: https://hgdownload.soe.ucsc.edu/goldenPath/hg38/bigZips/hg38.fa.gz. Gencode v36 gene annotation used in this study is available at https://ftp.ebi.ac.uk/pub/databases/gencode/Gencode_human/release_36/gencode.v36.annotation.gtf.gz. All additional information will be made available upon reasonable request to the authors.

Research involving human participants, their data, or biological material

Policy information about studies with human participants or human data. See also policy information about sex, gender (identity/presentation), and sexual orientation and race, ethnicity and racism.

Reporting on sex and gender	Patient sex is by default female as only patients with ovarian cancer were elligible. No gender information was disclosed.
Reporting on race, ethnicity, or other socially relevant groupings	There was no self-reporting in the study. Patients were selected based on medical assessment only.
Population characteristics	Primary chemo-naive high-grade serous ovarian cancer patients between age 71-86
Recruitment	Patients with high-grade primary or recurrent adenocarcinoma of ovarian, tubal, or peritoneal origin at FIGO stage III/IV and with no previous treatment line were eligible.
Ethics oversight	The use of material for research purposes was approved by the corresponding Swiss cantonal ethics commission (EKNZ: 2017-01900) and informed consent was obtained for all human primary material.

Note that full information on the approval of the study protocol must also be provided in the manuscript.

Field-specific reporting

Please select the one below that is the best fit for your research. If you are not sure, read the appropriate sections before making your selection.

☒ Life sciences ☐ Behavioural & social sciences ☐ Ecological, evolutionary & environmental sciences

For a reference copy of the document with all sections, see nature.com/documents/nr-reporting-summary-flat.pdf

Life sciences study design

All studies must disclose on these points even when the disclosure is negative.

Sample size	No statistical method was used to predetermine sample size. This study is a pilot study to demonstrate the potential of single-cell long-reads sequencing in oncology. We have 5 samples derived from 3 patients with high-grade serous ovarian cancer. The number of patients was limited to secure sufficient sequencing depth. 2500 cells were sequenced, ~500 per patient.
Data exclusions	No data was excluded.
Replication	Independent replication on more patients was not available. Validation was performed on independent samples from the same patients. Findings were intended to be patient specific.
Randomization	n/a
Blinding	n/a

Reporting for specific materials, systems and methods

We require information from authors about some types of materials, experimental systems and methods used in many studies. Here, indicate whether each material, system or method listed is relevant to your study. If you are not sure if a list item applies to your research, read the appropriate section before selecting a response.

Materials & experimental systems

n/a	Involved in the study
☐	☒ Antibodies
☒	☐ Eukaryotic cell lines
☒	☐ Palaeontology and archaeology
☒	☐ Animals and other organisms
☒	☐ Clinical data
☒	☐ Dual use research of concern
☒	☐ Plants

Methods

n/a	Involved in the study
☒	☐ ChIP-seq
☒	☐ Flow cytometry
☒	☐ MRI-based neuroimaging

Antibodies

Antibodies used

The following antibodies were used for this study: rabbit IGF2BP2 (C-terminal-specific, ThermoFisher Scientific, cat. no. MA5-42874), EpCAM (Cell Signaling Technologies, cat. no. 5488S,) and goat anti-rabbit Alexa Fluor® 647 (Cell Signaling Technology, cat. no. 4414).

Validation

rabbit IGF2BP2: "Immunohistochemistry analysis of IGF2BP2 in paraffin-embedded human colon. Samples were incubated with IGF2BP2 Monoclonal antibody (Product # MA5-42874) using a dilution of 1:100 (40x lens). Perform microwave antigen retrieval with 10 mM Tris/EDTA buffer pH 9.0 before commencing with IHC staining protocol." https://www.thermofisher.com/antibody/product/IGF2BP2-Antibody-clone-2O3W8-Recombinant-Monoclonal/MA5-42874
EpCAM: "This Cell Signaling Technology antibody is conjugated to Alexa Fluor® 555 fluorescent dye and tested in-house for immunofluorescent analysis in human cells. The antibody is expected to exhibit the same species cross-reactivity as the unconjugated EpCAM (VU1D9) Mouse mAb #2929. Dillution 1:200 - 1:400" https://www.cellsignal.com/products/antibody-conjugates/epcam-vu1d9-mouse-mab-alexa-fluor-555-conjugate/5488
goat anti-rabbit Alexa Fluor® 647: " Anti-rabbit IgG (H+L) F(ab')2 Fragment was conjugated to Alexa Fluor® 647 fluorescent dye under optimal conditions and formulated at 2 mg/ml. This F(ab')2 fragment product results in less non-specific binding, as it lacks the Fc domain that can bind to the cells with Fc receptors. Dillution 1:500 – 1:2000" https://www.cellsignal.com/products/secondary-antibodies/anti-rabbit-igg-h-l-f-ab-2-fragment-alexa-fluor-647-conjugate/4414

Plants

Seed stocks

n/a

Novel plant genotypes

n/a

Authentication

n/a
